# Assessing the Relationship of Patient Reported Outcome Measures With Functional Status in Dysferlinopathy: A Rasch Analysis Approach

**DOI:** 10.3389/fneur.2022.828525

**Published:** 2022-03-10

**Authors:** Anna G. Mayhew, Meredith K. James, Ursula Moore, Helen Sutherland, Marni Jacobs, Jia Feng, Linda Pax Lowes, Lindsay N. Alfano, Robert Muni Lofra, Laura E. Rufibach, Kristy Rose, Tina Duong, Luca Bello, Irene Pedrosa-Hernández, Scott Holsten, Chikako Sakamoto, Aurélie Canal, Nieves Sánchez-Aguilera Práxedes, Simone Thiele, Catherine Siener, Bruno Vandevelde, Brittney DeWolf, Elke Maron, Heather Gordish-Dressman, Heather Hilsden, Michela Guglieri, Jean-Yves Hogrel, Andrew M. Blamire, Pierre G. Carlier, Simone Spuler, John W. Day, Kristi J. Jones, Diana X. Bharucha-Goebel, Emmanuelle Salort-Campana, Alan Pestronk, Maggie C. Walter, Carmen Paradas, Tanya Stojkovic, Madoka Mori-Yoshimura, Elena Bravver, Jordi Díaz-Manera, Elena Pegoraro, Jerry R. Mendell, Volker Straub

**Affiliations:** ^1^The John Walton Muscular Dystrophy Research Centre, Translational and Clinical Research Institute, Newcastle University and Newcastle Hospitals NHS Foundation Trust, Newcastle upon Tyne, United Kingdom; ^2^Center for Translational Science, Division of Biostatistics and Study Methodology, Children's National Health System, Washington, DC, United States; ^3^Pediatrics, Epidemiology and Biostatistics, George Washington University, Washington, DC, United States; ^4^The Abigail Wexner Research Institute at Nationwide Children's Hospital, Columbus, OH, United States; ^5^The Jain Foundation, Seattle, WA, United States; ^6^The Children's Hospital at Westmead, The University of Sydney, Sydney, NSW, Australia; ^7^Cooperative International Neuromuscular Research Group (CINRG), Children's National Health System, Washington, DC, United States; ^8^Lucile Salter Packard Children's Hospital at Stanford, Neurology, Palo Alto, CA, United States; ^9^Department of Neuroscience, University of Padova, Padua, Italy; ^10^Physical Medicine and Rehabilitation, Hospital de la Santa Creu i Sant Pau, Barcelona, Spain; ^11^Neuroscience Institute, Carolinas Neuromuscular/ALS-MDA Center, Carolinas HealthCare System, Charlotte, NC, United States; ^12^Department of Physical Rehabilitation, National Center Hospital, National Center of Neurology and Psychiatry Tokyo, Tokyo, Japan; ^13^Institut de Myologie, AP-HP, GH Pitié-Salpêtrière, Paris, France; ^14^Neurorehabilitation Unit, Rehabilitation Hospital Universitario Virgen del Rocío Sevilla, Seville, Spain; ^15^Department of Neurology, Friedrich-Baur-Institute, Ludwig-Maximilians-University of Munich, Munich, Germany; ^16^Department of Neurology, Washington University School of Medicine, St. Louis, MO, United States; ^17^Service des Maladies Neuromusculaire et de la SLA, Hôpital de La Timone, Marseille, France; ^18^ELAN-PHYSIO, Praxis für Physiotherapie Maron, Berlin, Germany; ^19^Magnetic Resonance Centre, Institute for Cellular Medicine, Newcastle University, Newcastle upon Tyne, United Kingdom; ^20^AIM & CEA NMR Laboratory, Institute of Myology, Pitié-Salpêtrière University Hospital, Paris, France; ^21^Charite Muscle Research Unit, Experimental and Clinical Research Center, A Joint Cooperation of the Charité Medical Faculty and the Max Delbrück Center for Molecular Medicine, Berlin, Germany; ^22^Department of Neurology and Neurological Sciences, Stanford University School of Medicine, Stanford, CA, United States; ^23^Department of Neurology Children's National Health System, Washington, DC, United States; ^24^National Institutes of Health (NINDS), Bethesda, MD, United States; ^25^Neuromuscular Unit, Department of Neurology, Hospital U. Virgen del Rocío/Instituto de Biomedicina de Sevilla, Sevilla, Spain; ^26^Department of Neurology, National Center Hospital, National Center of Neurology and Psychiatry Tokyo, Tokyo, Japan; ^27^Centro de Investigación Biomédica en Red en Enfermedades Raras (CIBERER), Barcelona, Spain; ^28^Neuromuscular Disorders Unit, Neurology Department, Hospital de la Santa Creu i Sant Pau, Barcelona, Spain

**Keywords:** limb girdle muscular dystrophy, dysferlinopathy, PROMs, quality of life, clinical outcome assessments

## Abstract

Dysferlinopathy is a muscular dystrophy with a highly variable functional disease progression in which the relationship of function to some patient reported outcome measures (PROMs) has not been previously reported. This analysis aims to identify the suitability of PROMs and their association with motor performance.Two-hundred and four patients with dysferlinopathy were identified in the Jain Foundation's Clinical Outcome Study in Dysferlinopathy from 14 sites in 8 countries. All patients completed the following PROMs: Individualized Neuromuscular Quality of Life Questionnaire (INQoL), International Physical Activity Questionnaire (IPAQ), and activity limitations for patients with upper and/or lower limb impairments (ACTIVLIMs). In addition, nonambulant patients completed the Egen Klassifikation Scale (EK). Assessments were conducted annually at baseline, years 1, 2, 3, and 4. Data were also collected on the North Star Assessment for Limb Girdle Type Muscular Dystrophies (NSAD) and Performance of Upper Limb (PUL) at these time points from year 2. Data were analyzed using descriptive statistics and Rasch analysis was conducted on ACTIVLIM, EK, INQoL. For associations, graphs (NSAD with ACTIVLIM, IPAQ and INQoL and EK with PUL) were generated from generalized estimating equations (GEE). The ACTIVLIM appeared robust psychometrically and was strongly associated with the NSAD total score (Pseudo *R*^2^ 0.68). The INQoL performed less well and was poorly associated with the NSAD total score (Pseudo *R*^2^ 0.18). EK scores were strongly associated with PUL (Pseudo *R*^2^ 0.69). IPAQ was poorly associated with NSAD scores (Pseudo *R*^2^ 0.09). This study showed that several of the chosen PROMs demonstrated change over time and a good association with functional outcomes. An alternative quality of life measure and method of collecting data on physical activity may need to be selected for assessing dysferlinopathy.

## Introduction

Dysferlinopathy is a rare, autosomal recessive muscular dystrophy caused by mutations in the *DYSF* gene, which encodes the skeletal muscle protein dysferlin (1, 2). The most common clinical diagnoses associated with dysferlinopathy are limb girdle muscular dystrophy type 2B (LGMDR2 dysferlin related) and a distal posterior myopathy known as Miyoshi myopathy 1 MM1 (3, 4). Though onset typically occurs during young adulthood, clinical presentation is inconsistent, with a wide range of ages of onset, patterns of muscle weakness, and severity, despite the fact that most patients share a loss of expression of the dysferlin protein (1, 5–7). Likewise, disease progression is variable; loss of ambulation occurs 5 to 35 years after onset of muscle weakness, while a small number of patients remain only mildly affected for decades (8, 9). A number of factors that may influence the clinical phenotype and progression of dysferlinopathy have been proposed, including exercise and the specific mutation (5, 10, 11), though no clear pattern of decline or genotype–phenotype correlation has been established.

Of key importance to developing appropriately designed clinical trials in this group and to provide support and appropriate management for patients is understanding the natural history of the disease over time, particularly its impact on function, activities of daily living and ultimately quality of life. These are generally described as Patient Reported Outcome Measures (PROMs) or Patient Centered Outcome Measures (PCOMs). They are deemed a necessity for evaluating disease progression and the efficacy and clinical meaningfulness of therapeutic interventions by experts (12) and regulatory authorities (13). Although some work has gone into examining PROMs in this group over time and has shown the ACTIVLIM to detect change even in a period of 6 months (14), limited work has been done on the other patient measures.

The Jain Clinical Outcomes Study (COS) for dysferlinopathy was established to address the lack of comprehensive natural history data for dysferlinopathy, particularly around PROMs, and to identify if these were associated with functional changes related to disease progression. The aim of the present paper is to evaluate the suitability and change of selected PROMs from baseline up to a maximum of 4 years and to examine the association between these measures and the North Star Assessment for Limb Girdle Type Muscular Dystrophies (NSAD) (15) and the Performance of Upper Limb (PUL) (16), both as measures of motor performance.

## Methods

Clinical Outcomes Study is a multicenter, international study of patients with a confirmed diagnosis of dysferlinopathy. Detailed study methods have been published previously (8, 14). In order to be included in COS, patients were required to have two pathogenic mutations in *DYSF*, or one pathogenic mutation plus either absent dysferlin expression on immunoblot or <20% dysferlin monocyte expression. Each participating site received local ethics approval and written informed consent was obtained for all patients. The study was registered at ClinicalTrials.gov (NCT01676077).

The present study includes data from 204 patients at 14 sites; one original site was excluded from the present analysis due to the level of missing data over the 4-year period. Participants were evaluated on PROMs up to five times over the course of the study: baseline (visit 1), 12 (V2), 24 (V3), 36 (V4), and for some at 48 (V5) months. Visits took place between November 2012 and March 2018. Follow-up rate was high with 163 participants (87.2%) completing all five study visits across the 14 sites.

### Demographic and Clinical Measures

Self-reported race (if allowed by the participating country) and patient gender were recorded at baseline. Age at each study visit was approximated using the visit date and patient's year of birth. Clinical diagnosis of LGMDR2, MM1, or other (consisting primarily of hyperCKemia) was recorded based on the diagnostic label given by the diagnosing clinician, as reported by patients. Ambulatory status was defined at the point of assessment by the ability to complete a 10-m walk distance independently (gait aids permissible) as part of the physiotherapy assessment.

### Outcome Measures

A wide range of measures were included in the study, including measures of function and muscle strength, in order to establish their usefulness in assessing disease progression over time. For this study, we examined all patients who completed the following PROMs as appropriate: ACTIVLIM, International Physical Activity Questionnaire (IPAQ), and the Individualized Neuromuscular Quality of Life Questionnaire (INQoL). Nonambulant patients also completed the Egen Klassifikation (EK) scale. Assessments were conducted annually at baseline, years 1, 2, and 3 for some at year 4. Data were also collected on motor performance at these time points (8).

The ACTIVLIM is a validated patient reported outcome measure of functional ability based on perceived difficulty in performing specific activities of daily living (17). Total scores range from 0 to 36, with higher scores suggesting better function.

International Physical Activity Questionnaire assesses a person's physical activity levels using a set of seven questions and describes that activity in terms of time spent and level of intensity (18). There are two forms of output from scoring the IPAQ. Results can be reported in categories (low activity levels, moderate activity levels or high activity levels) or as a continuous variable (Metabolic Equivalent Task (MET) minutes a week).

Individualized Neuromuscular Quality of Life Questionnaire is a quality of life measure that aims to capture the impact of key muscle disease symptoms and the impact on activities, independence, relationships, and body image (19).

The EK scale is a patient's assessment of their “own functioning” related to daily tasks and was designed for nonambulant pediatric and adult patients with Duchenne muscular dystrophy (DMD) and spinal muscular atrophy (20, 21).

The NSAD is a Rasch-developed clinician reported outcome (ClinRO) of motor performance suitable for ambulant and nonambulant adults and children, validated in this dysferlinopathy (LGMDR2) cohort, and has been shown to be very sensitive to change over 1 year (15).

The PUL scale was designed specifically to measure upper limb motor performance across the spectrum of severity in pediatric and adult DMD, is also a Rasch-built ClinRO, and has been shown to be valid and sensitive to change in DMD over time (22, 23).

### Rasch Analysis

For rating scales (ACTIVLIM, INQoL, EK), Rasch analysis (RUMM2030 software) was conducted. Essentially, a Rasch analysis examines the extent to which the observed data (in this instance, patients' ratings on scale items) “fit” with predictions of those ratings from the Rasch model (which defines how a set of items should perform to generate discriminant, reliable, and valid measurements) (24). Data were entered into Rasch Unidimensional Measurement Model RUMM2030 (standard version; RUMM Laboratory Pty Ltd, Perth, Australia). Analyses assessed (1) item fit to the underlying construct of the questionnaire (all items should lie within a fit residual range of SD 2.5); (2) whether the scoring within each item reflected perceived progression (ordering of thresholds); (3) reliability, as indicated by a Person Separation Index of more than 0.8, which is equivalent to Cronbach's alpha (25); (4) targeting of the scales by plotting item location for them separately and comparing individual person–item threshold distribution maps for any significant floor or ceiling effects or gaps in the continuum of measurements in the two scales; (5) dependency, which determines if the response on one item influences the response on another item (this is assessed by examining the residual correlations); and finally (6) a *t*-test of unidimensionality. Details on these methods can be found elsewhere (26).

### Statistical Analysis

Data were analyzed using descriptive statistics for each visit and the entire study. For associations, graphs (NSAD with ACTIVLM, IPAQ and INQoL and EK with PUL) were generated from generalized estimating equations (GEE) based on preliminary examination of cubic spline graphs. Spearman correlation coefficients are presented for reference. Pseudo *R*^2^ for fixed effects were estimated from generalized linear mixed models using techniques described by Jaeger et al. (27).

## Results

Of the cohort of 204 patients, 97 were men and 107 were women. Median age at baseline assessment was 36 years of age, range of 8–86. At baseline 76% of the study cohort were ambulant.

### Descriptive Results

For ACTIVLIM, a total of 733 questionnaires were reviewed over 4 time points (787 for Rasch over 5 time points); 2% showed scores under 16 and were excluded from Rasch analysis as they would have answered a different subset of four items. Median score was 23 (range 0–36).

For IPAQ, a total of 748 questionnaires were reviewed over 4 time points. Eighty-two percent of patients undertook no vigorous exercise, 59% undertook no moderate exercise, 40% did no walking exercise for more than 10 min. The mean time spent sitting per day was 9.4 h (SD 4.7). Twenty-four percent were nonambulant.

For INQoL, a total of 660 questionnaires were reviewed over 4 time points. Total scores were calculated on a subset of items not including the treatment section, as only 23% of the cohort answered the questions relating to treatment and their responses included a wide variety of management techniques which were not comparable. However, a sub-total score was calculated on all questions unrelated to treatment.

For EK, a total of 208 questionnaires were reviewed over 4 time points (245 for Rasch over 5 time points) in nonambulant individuals. The median score was 7 (range 1–21), out of a maximum of 30, with higher scores indicating being less able.

See Appendix A for a summary of PROM data over the different time points.

### Rasch Analysis

**Figure 1:**
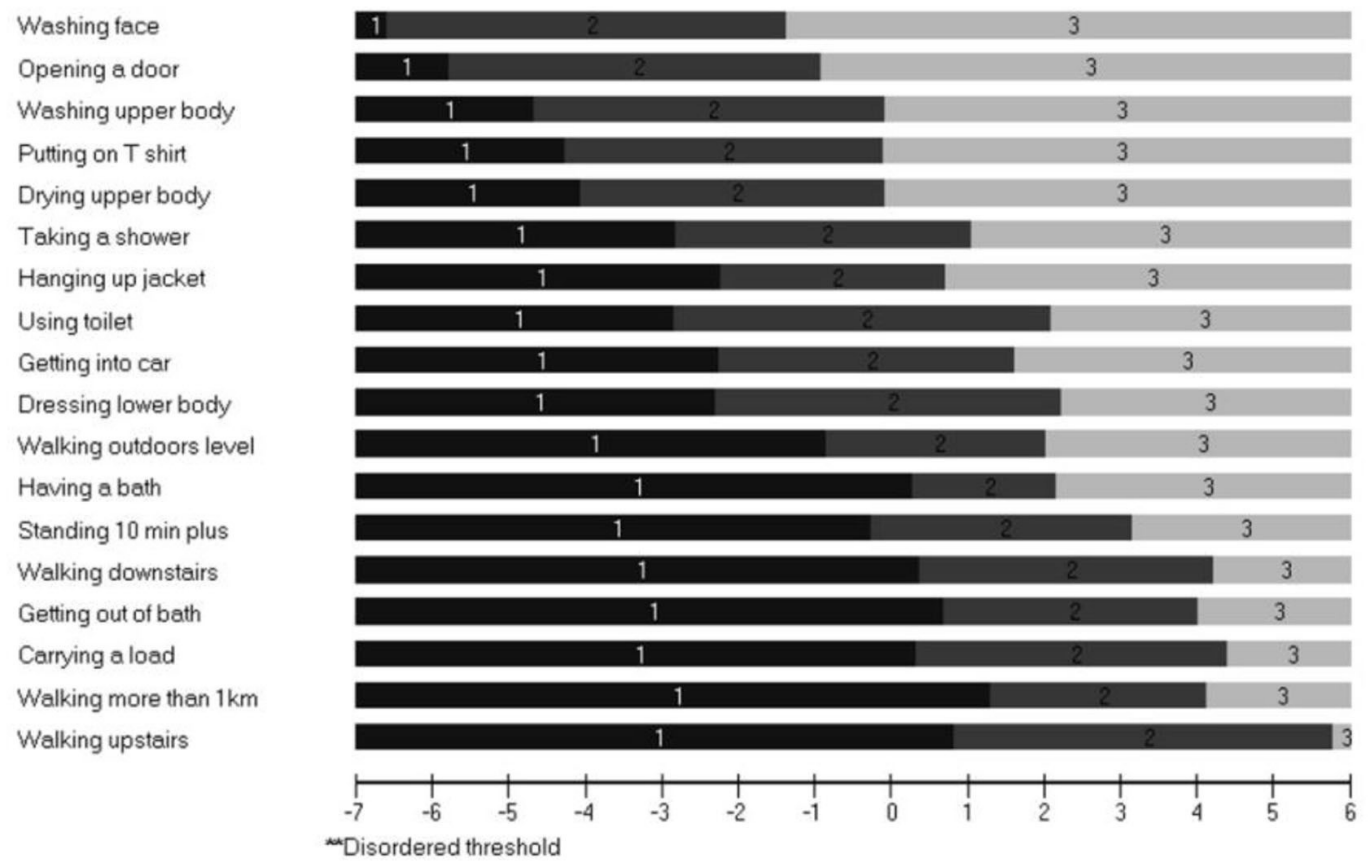
Threshold map showing all items ordered in the ACTIVLIM Scale in order of difficulty. Threshold map for items in ranked order of difficulty according to Rasch analysis. “Impossible” labeled 1; “With difficulty” labeled 2; “Easy” labeled 3. There were no reversed thresholds. This is why the figure legend suggested that disordered thresholds will be labeled with a double asterisk but none are disordered. The implication of this is that as a person's functional ability increases (move left to right) they would be more likely to score higher in a consistent and hierarchical way for all questions.

The results of the Rasch analysis for the three rating scales are presented in Table 1. The data included the year 4 data that were available for these Rasch analyses. The ACTIVLIM presented as a scale with good fit to the population with all items showing ordered thresholds (Figure 1) and 89% of items showing good fit with minimal dependency and an acceptable level of unidimensionality.

**Table 1:** Summary Rasch results for ACTIVLIM, INQoL, and EK scales.

	**Rasch summary**	**Item fit**	**Person fit**		**Reliability**	**Item fit**		**Dependency**	**Test of unidimensionality**
	N (no. of extremes) available	Mean (SD)	Mean (SD)	Overall item-trait interaction chi 2 value (DF)	Psi with extremes	Ordered thresholds	Number of items with good fit*	Number of pairs of dependent items	Acceptable?
ACTIVLIM	787 (66) = 721	−0.05 (1.963)	−0.37 (0.883)	0.00 (162)	0.95	18/18 (100 %)	16/18 (89%) 16^a^ / 16^b^	2	Not acceptable
INQoL	660 (2) = 658	0.65 (3.039)	−0.09 (1.703)	0.00 (351)	0.95	15/39 (38%)	20/39 (51%) 26^a^ / 24^b^	33	Not acceptable
EK	250 (5) = 245	−0.22 (1.737)	−0.423 (0.576)	0.00 (120)	0.76	3/10 (30%)	8/10 (80%) 8^a^ / 9^b^	0	Acceptable

* *Fit: defined as fit residual inside the recommended range (–2.50 to +2.50) ^a^ and nonsignificant v2 probability (p < 0.001) ^b^*.

**Figure 2:**
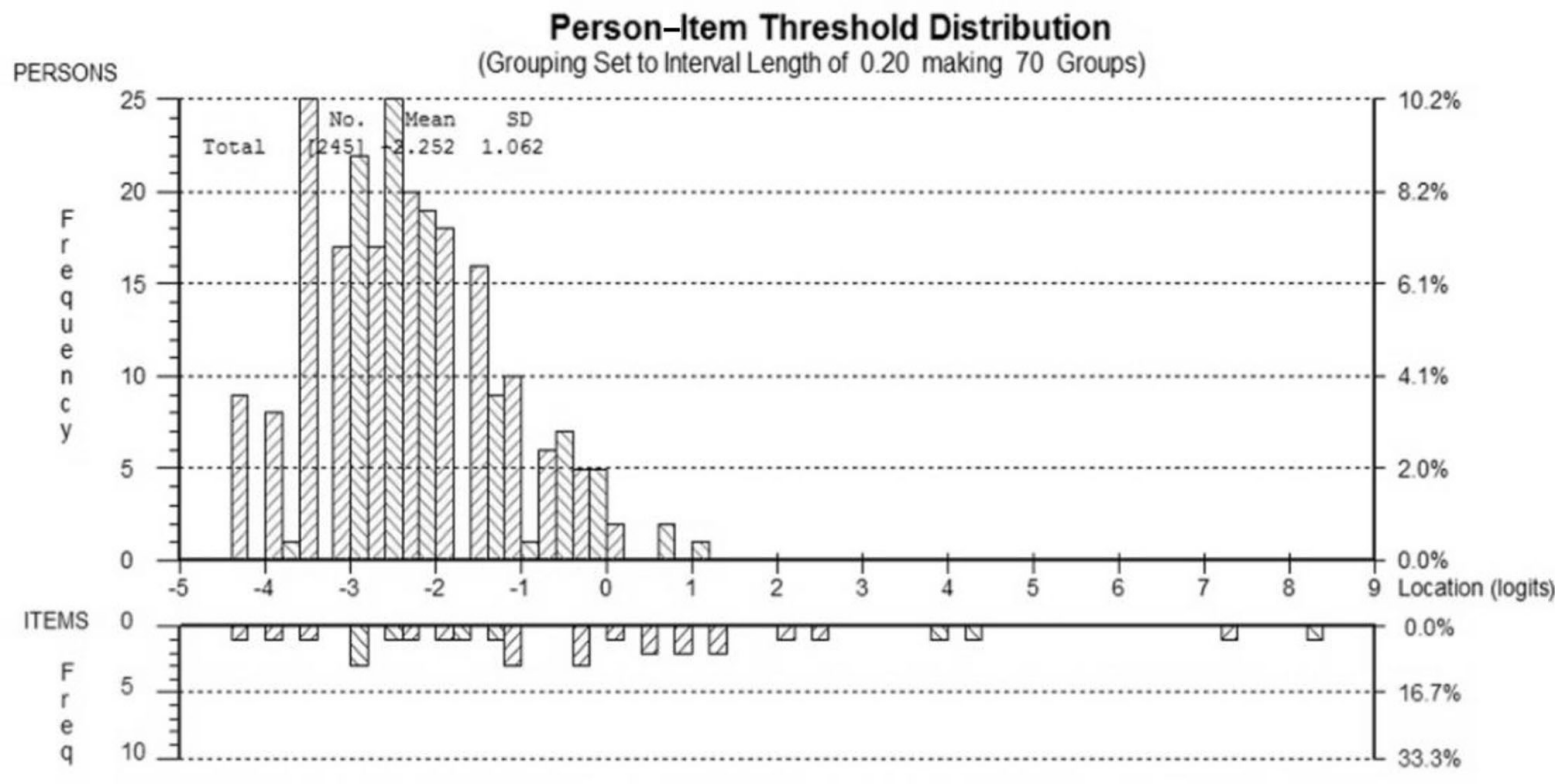
Person–item location distribution for the EK scale. Targeting of the patient sample (top) to the EK items (bottom). The figure shows the adequate targeting between the distribution of person measurements (upper histogram) and the distribution of item locations (lower histogram). There are no ceiling/floor effects indicated by the range of the person measurements (upper histogram “blocks”) falling within the item locations (lower histogram “blocks”).

The INQoL performed less well with poor ordering of thresholds (38%), unacceptable unidimensionality and limited item fit to the construct of Health Related Quality of Life (HRQoL), although adequate targeting was apparent with only a small floor effect. For the EK, ordering of the thresholds was low but items showed good fit and targeting (Figure 2) and unidimensionality was acceptable.

**Figure 3:**
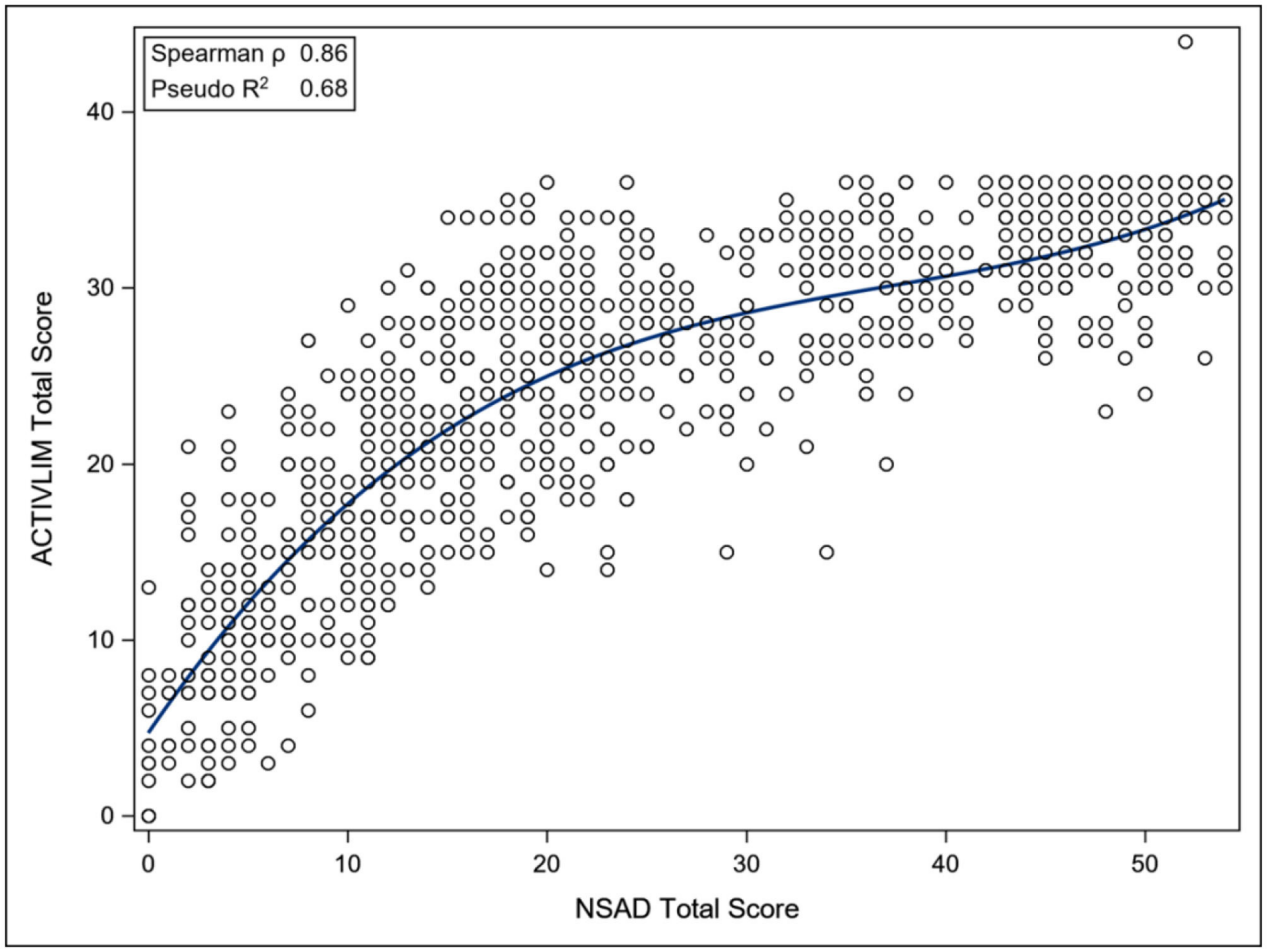
Association between Total NSAD score and ACTIVLIM. NSAD, North Star Assessment for Limb Girdle Type Muscular Dystrophies; ACTIVLIM, Measure of Activity Limitations in Daily Living.

**Figure 4:**
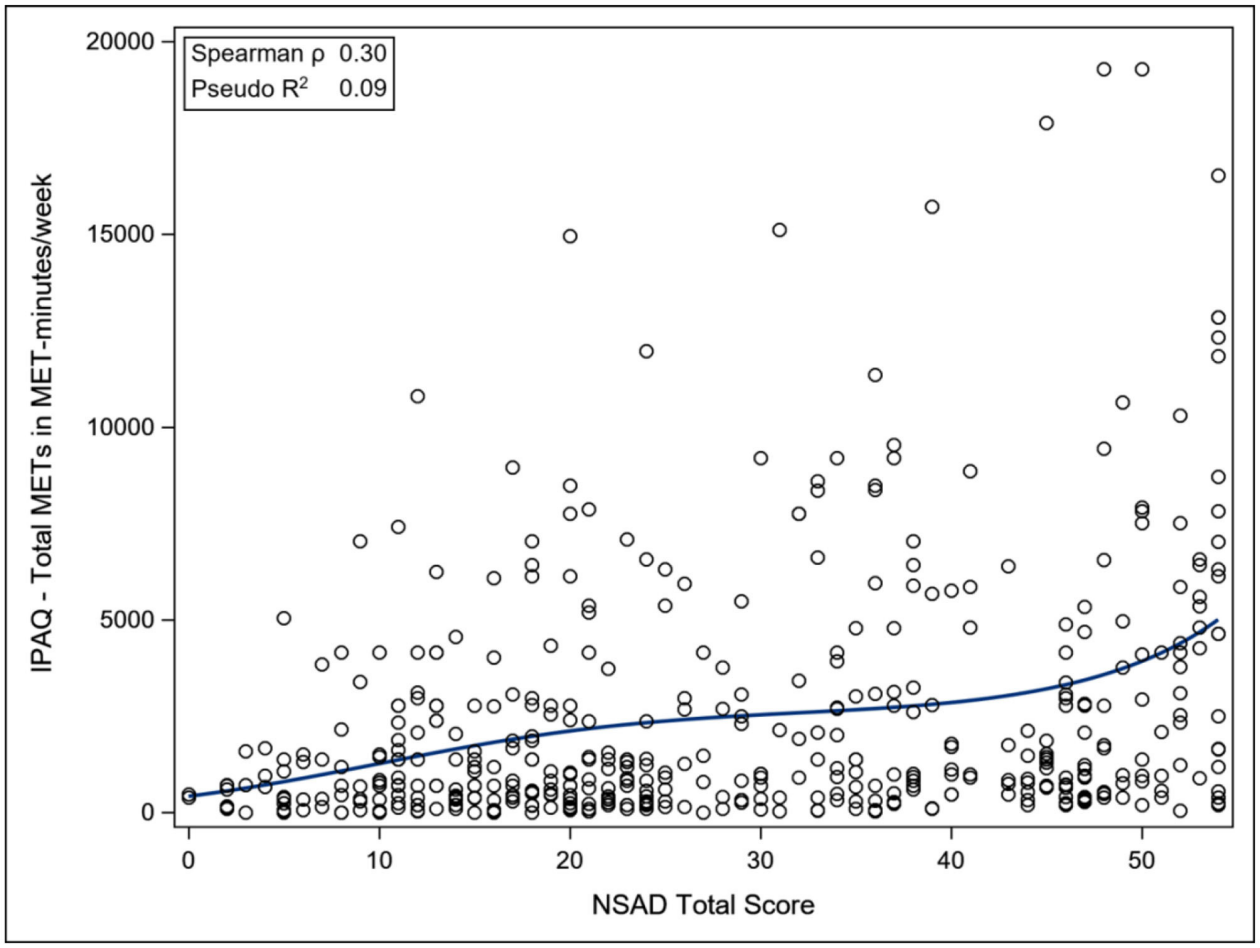
Association between total NSAD score and IPAQ. NSAD, North Star Assessment for Limb Girdle Type Muscular Dystrophies; IPAQ, International Physical Activity Questionnaire.

**Figure 5:**
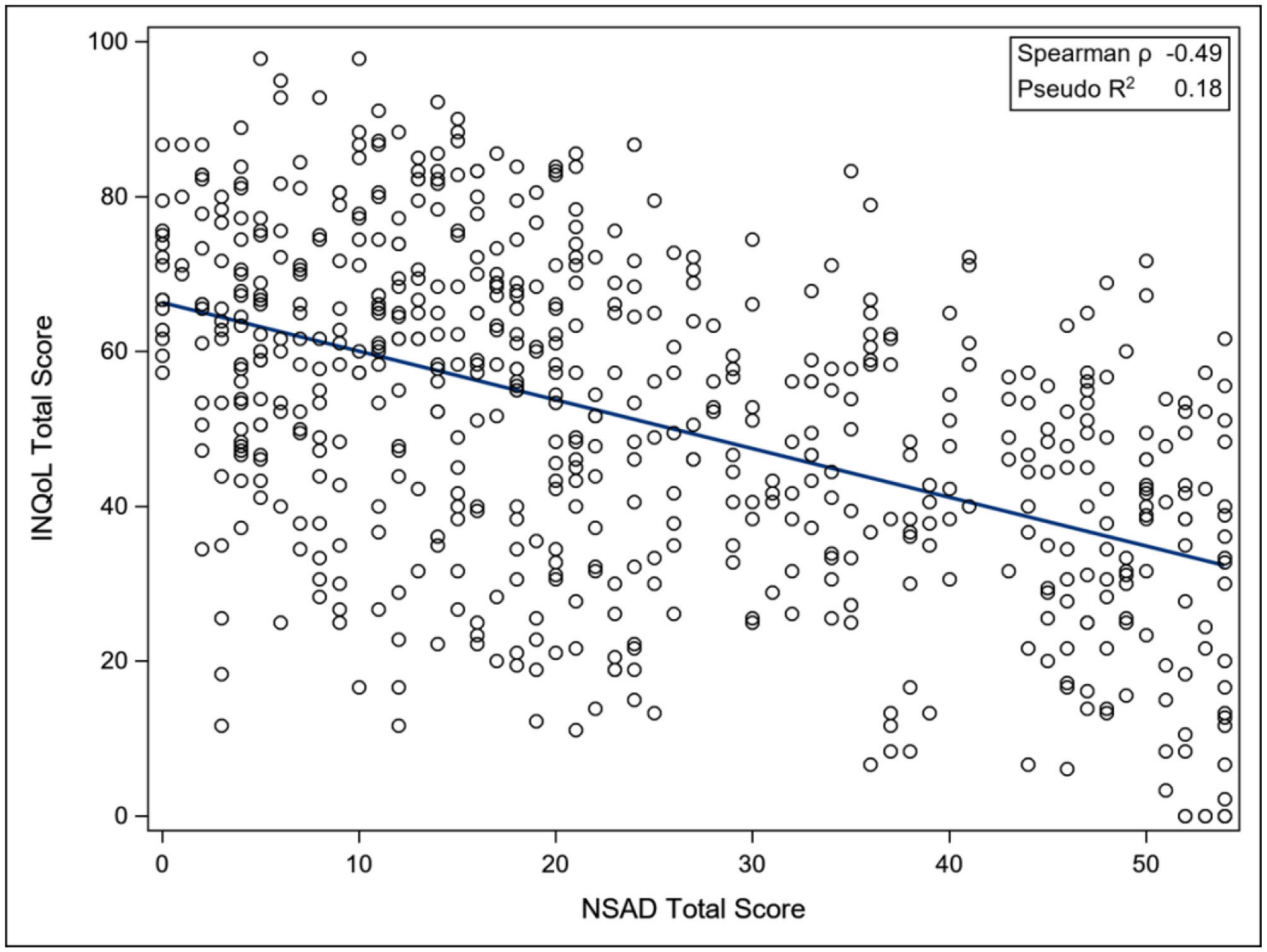
Association between total NSAD score and INQoL. NSAD, North Star Assessment for Limb Girdle Type Muscular Dystrophies; INQoL, Individualized Neuromuscular Quality of Life Questionnaire.

**Figure 6:**
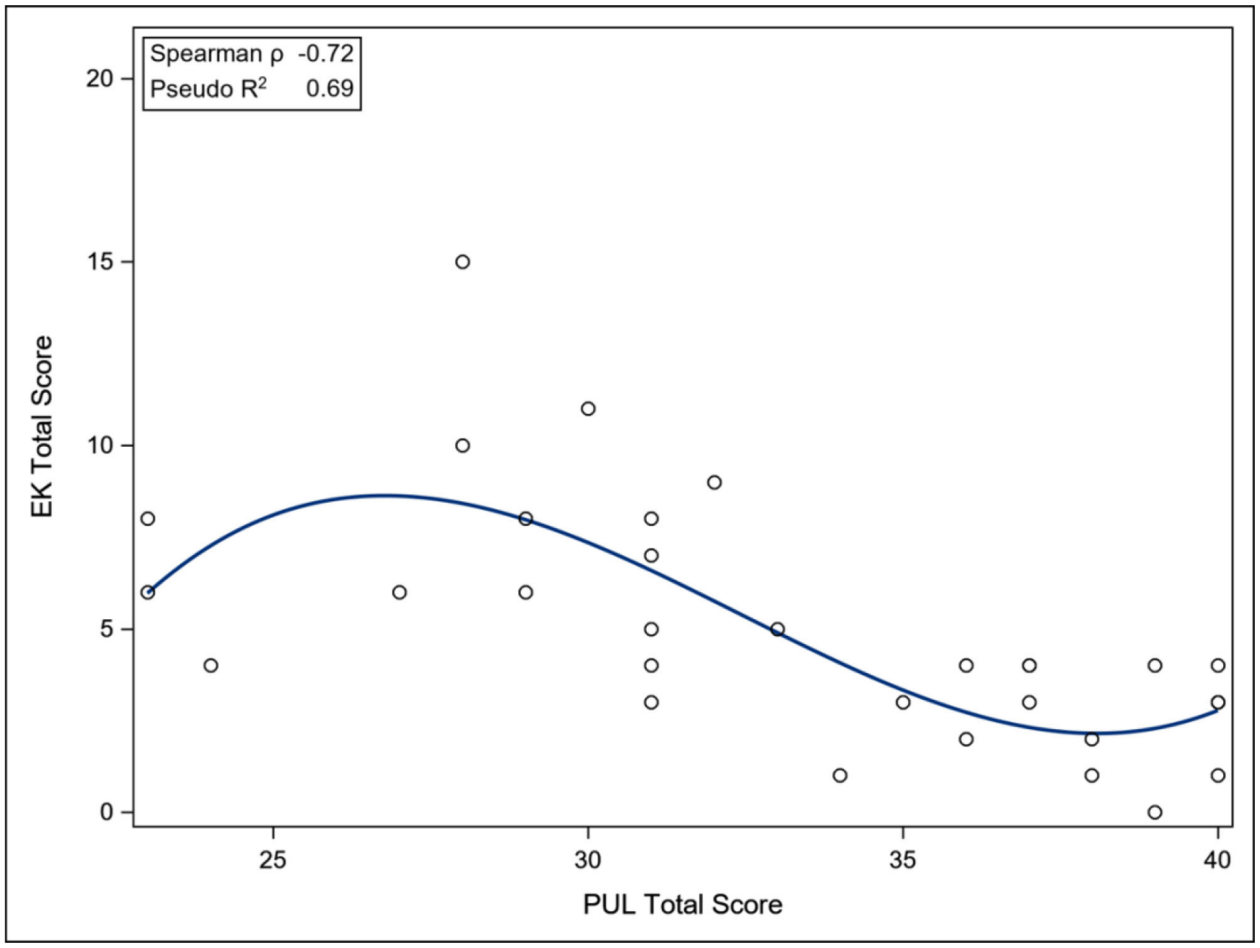
Association between total PUL score and EK scale. EK, Egen Klassification questionnaire; PUL, Performance of Upper Limb Scale.

The ACTIVLIM was strongly associated with the NSAD total score (Pseudo *R*^2^ 0.68), whereas the INQoL was poorly associated with the NSAD total score (Pseudo *R*^2^ 0.18), as was the IPAQ Total METS (Pseudo *R*^2^ 0.09). For the nonambulant population, the EK was strongly associated with the PUL total score (Pseudo *R*^2^ 0.69) (Figures 3–6).

## Discussion

Findings from the present study in dysferlinopathy provide useful information on the suitability (i.e., do the scales work as measurement tools?) of four PROMs to capture the patient's opinion. In addition, understanding the relationship between patient-centered outcomes with measures of motor performance can add clinical meaning to motor measures which in turn could be valuable within the clinical trial setting. For example, do changes in arm function measured on the PUL reflect changes in how easy or hard it is for an individual to wash and dress their upper body?

At the beginning of COS, the choice of PROMs needed careful consideration as no gold standard existed for this population. The ACTIVLIM and INQoL were chosen as they had been built using modern psychometric methods and had demonstrated some evidence of suitability in neuromuscular disease (17, 19). The IPAQ was used because activity had not been studied in the group and was felt to be a significant factor, and the EK was selected to capture “own functioning” in the nonambulant patient population. The ACTIVLIM performed particularly well psychometrically in this population with adequate targeting, ordered thresholds, and most items fitting the construct well. The EK scale contained several clinically relevant items for this group; however, some items around respiratory insufficiency appeared to be not relevant for the nonambulant dysferlinopathy patients. However, the association of arm function with scores on the EK has been demonstrated in DMD (28) and it may be that a subset of the items could be identified to capture function in the weaker population. The association of EK to arm function, although strong, is not linear, which may indicate that different domains are captured by the individual scales. The IPAQ also did not correlate with functional status as measured by NSAD; however, it is not designed specifically to capture activity and exercise in the nonambulant population and meta-analysis has shown that it is perhaps not capturing activity satisfactorily (18). The INQoL performed less well psychometrically, with poor ordering of thresholds and limited item fit to the construct of HRQoL, although adequate targeting was apparent with only a small floor effect. It was not associated with functional status, although it is understood that quality of life is not solely dependent on functional abilities (29) and many individuals with motor impairment report high satisfaction with their lives.

This study had some limitations. Not all the measures selected were suitable for nonambulant individuals, which made up a quarter of our cohort, which, for example, could result in a limited understanding of activity levels in this group. It may have been beneficial to review and adapt some of the PROMs prior to testing in such a large cohort. This could have identified any unnecessary or unsuitable questions: for example, the swallowing and eating items within the EK scale. However, evidence of how scales work in their original format removes the concern that changes made to it prior to full evaluation were unjustified. We also acknowledge that although chronic pain is recognized as being a significant issue for LGMD (30), we did not specifically evaluate this area using a chronic pain questionnaire although patients were asked about pain during their medical assessment.

The results of this analysis have benefited the design of an extension to this current study, where the quality of life measure has been replaced. More work needs to be undertaken to understand function in relationship to these new PROMs and to evaluate if change in function over time is reflected in the related PROM. Further work can also commence on refinement of some of the current tools such as the EK scale and IPAQ.

## Data Availability Statement

The raw data supporting the conclusions of this article will be made available by the authors, without undue reservation.

## Ethics Statement

The studies involving human participants were reviewed and approved by NRES Committee, North East-Newcastle & North Tyneside 2. Written informed consent to participate in this study was provided by the participants' legal guardian/next of kin.

## Author Contributions

AM, MKJ, VS, LL, LA, and LR: conceptualization of the project, methodology design, research implementation, results analysis, and manuscript writing. UM, HS, JF, RM, KR, and TD: research implementation, results analysis, and manuscript writing. MJ: results analysis and manuscript writing. HG: methodology design, research implementation, results analysis, and manuscript writing. HH: methodology design, research implementation, and manuscript writing. NS-A, ST, CSi, BV, BD, EM, MG, J-YH, AB, PC, SS, JD, KJ, DB-G, ES-C, AP, MW, CP, TS, MM-Y, EB, JD-M, EP, JM, LB, IP-H, SH, CSa, and AC: research implementation and manuscript writing. All authors contributed to the article and approved the submitted version.

## Funding

The estimated US $4 million needed to fund this study was provided by the Jain Foundation (www.jainfoundation.org). The John Walton Muscular Dystrophy Research Centre is part of the MRC Centre for Neuromuscular Diseases (Grant Number MR/K000608/1).

## Conflict of Interest

MJ receives fee support for PhD studies from the Jain Foundation. The remaining authors declare that the research was conducted in the absence of any commercial or financial relationships that could be construed as a potential conflict of interest.

## Publisher's Note

All claims expressed in this article are solely those of the authors and do not necessarily represent those of their affiliated organizations, or those of the publisher, the editors and the reviewers. Any product that may be evaluated in this article, or claim that may be made by its manufacturer, is not guaranteed or endorsed by the publisher.
