# Supplementary material for: Assessing the Relationship of Patient Reported Outcome Measures With Functional Status in Dysferlinopathy: A Rasch Analysis Approach
Source: Front Neurol. 2022 Mar 10;13:828525. doi: 10.3389/fneur.2022.828525 (PMC8961025; doi:10.3389/fneur.2022.828525)
Supplement: Supplementary file 1 [file Table_1.docx]

Appendix A

Table 1. Summary of PROM data over the different time points

|  | **Baseline** | **Year 1** | **Year 2** | **Year 3** | **Year 4** | **TOTAL for association analysis Baseline to Year 3** |
| --- | --- | --- | --- | --- | --- | --- |
| Total data points for IPAQ | 204 | 189 | 189 | 166 | None | 748 |
| % performing no vigorous exercise | 79.90% | 82.01% | 83.07% | 83.13% | - | 82.22% |
| % performing no moderate exercise | 54.41% | 56.61% | 60.85% | 65.66% | - | 59.09% |
| Number of data sets of EK data | 47 | 50 | 56 | 55 | (+37 for Rasch only) | 208 |
| Median (range) of EK scale | 8 (1-21) | 7 (1-20) | 7 (1-18) | 7 (1-20) | - | 7 (1-21) |
| Number of data sets of ACTIVLIM data | 200 | 189 | 185 | 159 | (+54 for Rasch only) | 733 |
| Median (range) for ACTIVLIM | 26 (0-36) | 23 (0-36) | 22 (0-36) | 20 (0-36) |  | 23 (0-36) |
| Number of data sets of INQoL data | 197 | 178 | 171 | 105 | 9 | 660 |
| Mean (SD) of INQoL based on raw data* | 49.9 (19.5) | 50.4 (20.5) | 53.0 (21.8) | 54.0 (20.7) | 56.0 (19.8) | 51.7 (20.6) |

**Does not include treatment section of the INQOL questionnaire*
