# Supplementary material for: Assessing the Relationship of Patient Reported Outcome Measures With Functional Status in Dysferlinopathy: A Rasch Analysis Approach
Source: Front Neurol. 2022 Mar 10;13:828525. doi: 10.3389/fneur.2022.828525 (PMC8961025; doi:10.3389/fneur.2022.828525)
Supplement: Supplementary file 2 [file Table_2.docx]

**Appendix B. Coinvestigators - The Jain COS Consortium**

**We acknowledge the work of the following members of the Consortium who have contributed to the collection of the data, but do not qualify for authorship on this paper.**

| **Name** | **Location** | **Role** | **Contribution** |
| --- | --- | --- | --- |
| Adrienne Arrieta MS | Children’s National Medical Center Washington, DC, USA | Lead Data Management Expert | Database development and training |
| Esther Hwang | Jain Foundation, Seattle, WA, USA | Patient Advocate | Development of study questionnaires, recruitment |
| Elaine Lee PhD | Jain Foundation, Seattle, WA, USA | Patient Advocate | Development of study questionnaires, recruitment |
| Isabel Illa MD | Hospital de la Santa Creu i Sant Pau/ CIBERER, Barcelona, Spain | Director of the Neuromuscular Unit | Study design |
| Eduard Gallardo MD | Hospital de la Santa Creu i Sant Pau/ CIBERER, Barcelona, Spain | Consultant Scientist | Confirmation of eligibility using monocyte assay |
| Izaskun Belmonte Jimeno PT | Servei de Medicina F ísica i Rehabilitació, Hospital de la Santa Creu i Sant Pau, Barcelona, Spain | Physiotherapist | Acquisition of physiotherapy data |
| Elena Montiel-Morillo | Servei de Medicina F ísica i Rehabilitació, Hospital de la Santa Creu i Sant Pau, Barcelona, Spain | Physiotherapist | Acquisition of physiotherapy data |
| Jaume Llauger Rossello | Radiology department, Universitat Autònoma de Barcelona, Hospital de la Santa Creu i Sant Pau, Barcelona, Spain | Radiologist | Collection and analysis of MRI data |
| Bruce Harwick | Department of Radiology, CMC Mercy Charlotte, Carolinas HealthCare System Neurosciences Institute, Charlotte, NC, USA | NMR Technologist | MRI data acquisition |
| Jackie Sykes RN, BSN | Carolinas HealthCare System, Charlotte, NC, USA | Study Coordinator | Study coordination and data management |
| Susan Sparks | Carolinas HealthCare System, Charlotte, NC, USA | Neurologist | Recruiting PI and PI for first three visits |
| Brent Yetter MS | Nationwide Children’s Hospital, Columbus, OH, USA | Study Coordinator | Study coordination and data management |
| Mark Smith MS, DABMP RT(MR) | Department of Radiology, Nationwide Children’s Hospital, Columbus, OH, USA | MRI Technologist | MRI data acquisition |
| Bernard Lapeyssonie PT | Neuromuscular and ALS center, La Timone Hospital, Aix-Marseille Université, Marseille, France | Physiotherapist | Acquisition of physiotherapy data |
| Bruno Vandevelde | Neuromuscular and ALS center, La Timone Hospital, Aix-Marseille Université, Marseille, France | Physiotherapist | Acquisition of physiotherapy data |
| David Bendahan PhD | Centre de Résonance, MagnétiqueBiologique et Médicale, UMR CNRS 7339, Marseille, France | MRI Lead | MRI technologist |
| Yann Le Fur PhD | Aix-Marseille Université, Marseille, France | MRI Technologist | MRI data acquisition |
| Attarian Shahram MD, PhD | Neuromuscular and ALS center, La Timone Hospital, Aix-Marseille Université, Marseille, France | Study Doctor | Medical history, medical and physical examination |
| Testot-Ferry Albane CRA | Neuromuscular and ALS center, La Timone Hospital, Aix-Marseille Université, Marseille, France | Study Coordinator | Study coordination and data management |
| Eva M. Coppenrath MD | Department of Clinical Radiology, Ludwig-Maximilians-University Munich, Germany | Radiologist | MRI data acquisition |
| Sabine Krause | Ludwig-Maximilians-University, Munich, Germany | Study Doctor | Medical history, medical and physical examination |
| Olivia Schreiber- Katz | Ludwig-Maximilians-University, Munich, Germany | Study Doctor | Medical history, medical and physical examination |
| Elizabeth Harris MD | The John Walton Muscular Dystrophy Research Centre, Newcastle upon Tyne, UK | Study Doctor | Medical history, medical and physical examination |
| Teresinha Evangelista MD | The John Walton Muscular Dystrophy Research Centre, Newcastle upon Tyne, UK | Study Doctor | Medical history, medical and physical examination |
| Alex Murphy MD | The John Walton Muscular Dystrophy Research Centre, Newcastle upon Tyne, UK | Study Doctor | Medical history, medical and physical examination |
| Michelle Eagle PhD | The John Walton Muscular Dystrophy Research Centre, Newcastle upon Tyne, UK | Physiotherapist | Study design, acquisition of physiotherapy data |
| Dionne Moat BSc | The John Walton Muscular Dystrophy Research Centre, Newcastle upon Tyne, UK | Physiotherapist | Acquisition of physiotherapy data |
| Jassi Amritpal Singh Sodhi BSc | The John Walton Muscular Dystrophy Research Centre, Newcastle upon Tyne, UK | Physiotherapist | Acquisition of physiotherapy data |
| Tim Hodgson MClinRES | Magnetic Resonance Centre, Newcastle University, UK | Radiologist | MRI data acquisition |
| Fiona E. Smith | Magnetic Resonance Centre, Newcastle University, UK, | NMR Scientist | MRI analysis and training |
| Ian Wilson | Magnetic Resonance Centre, Newcastle University, UK | NMR Scientist | MRI analysis |
| Dorothy Wallace BSc | Magnetic Resonance Centre, Institute for Cellular Medicine, Newcastle University, UK | Radiologist | MRI data acquisition |
| Louise Ward DCR | Magnetic Resonance Centre, Institute for Cellular Medicine, Newcastle University, UK | Radiologist | MRI data acquisition |
| Debra Galley | Magnetic Resonance Centre, Newcastle University, UK | Radiology Assistant | MRI data acquisition |
| Chiara Calore | University of Padova, Italy | Study Doctor | Medical history, medical and physical examination |
| Claudio Semplicini | University of Padova, Italy | Sub-PI | Acquisition of physiotherapy data medical history, medical and physical examination |
| Roberto Stramare MD | Radiology Unit, Department of Medicine, University of Padova, Italy | Radiologist | MRI data acquisition |
| Alessandro Rampado MRT | Radiology Unit, Department of Medicine, University of Padova, Italy | Radiologist | MRI data acquisition |
| Suna Turk MSc | AIM & CEA NMR Laboratory, Institute of Myology, Pitié-Salpêtrière University Hospital, 47-83, Paris, France | Radiologist | MRI data acquisition |
| Harmen Reyngoudt | AIM & CEA NMR Laboratory, Institute of Myology, Pitié-Salpêtrière University Hospital, 47-83, Paris, France | NMR Scientist | MRI training and analysis |
| Ericky Caldas | AIM & CEA NMR Laboratory, Institute of Myology, Pitié-Salpêtrière University Hospital, 47-83, Paris, France | NMR Scientist | MRI training and analysis |
| Cyrille Theis | Institut de Myologie, Paris, France | Radiologist | MRI data acquisition |
| Oumar Diabaté | Institut de Myologie, Paris, France | Study Coordinator | Study coordination and data management |
| Matthew Harms | Washington University, St. Louis, MO, USA | Co PI, Study Doctor | Recruitment, medical history, medical and physical examination |
| Julaine M. Florence | Washington University, St. Louis, MO, USA | Physiotherapist | Acquisition of physiotherapy data |
| Linda Schimmoeller | Washington University, St. Louis, MO, USA | Study Coordinator | Study coordination and data management |
| Glenn Foster RTR (MR) | Center for Clinical Imaging Research CCIR, Washington University, St. Louis, MO, USA | MRI Technologist | MRI data acquisition |
| Pilar Carbonell MD | Hospital U. Virgen del Rocío/Instituto de Biomedicina de Sevilla, Spain | Study Doctor | Medical history, medical and physical examination |
| Macarena Cabrera MD | Hospital U. Virgen del Rocío/Instituto de Biomedicina de Sevilla, Spain | Study Doctor | Medical history, medical and physical examination |
| Juan Bosco Mendez | Hospital U. Virgen del Rocío/Instituto de Biomedicina de Sevilla, Spain | Rehabilitation Doctor | Acquisition of physiotherapy data |
| Yolanda Morgado MD | Hospital U. Virgen de Valme/Instituto de Biomedicina de Sevilla, Spain | Study Doctor | Medical history, medical and physical examination |
| Susana Rico Gala MD | Department of Radiology, Hospital U. Virgen de Valme, Sevilla, Spain | Radiologist | MRI data acquisition |
| Jennifer Perez | Stanford University School of Medicine, Stanford, CA, USA | Study Coordinator | Study coordination and data management |
| Anne Marie Sawyer FSMRT | Lucas Centre for Imaging, Stanford University School of Medicine, Stanford, CA, USA | MRI Lead | MRI data acquisition |
| Carolina Tesi-Rocha | Stanford University School of Medicine, Stanford, CA, USA | Sub-I, Study Doctor | Medical history, medical and physical examination |
| Richard Gee | Stanford University School of Medicine, Stanford, CA, USA | Physiotherapist | Acquisition of physiotherapy data |
| Nigel F. Clarke MD | Institute for Neuroscience and Muscle Research, Sydney, Australia | Original PI. *Deceased.* | Recruitment and study design |
| Sarah Sandaradura MD | Institute for Neuroscience and Muscle Research, Sydney, Australia | Study Doctor | Acquisition of medical history, medical and physical assessments |
| Roula Ghaoui MD | Institute for Neuroscience and Muscle Research, Sydney, Australia | Study Doctor | Acquisition of medical history, medical and physical assessments |
| Kayla Cornett | Institute for Neuroscience and Muscle Research, Sydney, Australia | Physiotherapist | Acquisition of physiotherapy data |
| Claire Miller PT | Institute for Neuroscience and Muscle Research, Sydney, Australia | Physiotherapist | Acquisition of physiotherapy data |
| Sheryl Foster MHlthSc | Department of Radiology, Westmead Hospital; Faculty of Health Sciences, University of Sydney, Australia | Radiologist | MRI data acquisition |
| Anthony Peduto MBBS | Department of Radiology, Westmead Hospital; Faculty of Health Sciences, University of Sydney, Australia | Radiologist | MRI data acquisition |
| Noriko Sato MD PhD | Department of Radiology, National Center Hospital, National Center of Neurology and Psychiatry, Tokyo, Japan | Radiologist | MRI data acquisition |
| Takeshi Tamaru MRT | Department of Radiology, National Center Hospital, National Center of Neurology and Psychiatry, Tokyo, Japan | Radiologist | MRI data acquisition |
| Shin’ich Takeda | National Center of Neurology and Psychiatry, Tokyo | Director | Supervision of site investigators |
| Ai Ashida PT | National Center of Neurology and Psychiatry, Tokyo | Physiotherapist | Acquisition of physiotherapy data |
| Tatayuki Tateishi | National Center of Neurology and Psychiatry, Tokyo | Physiotherapist | Acquisition of physiotherapy data |
| Hiroyuki Yajima | National Center of Neurology and Psychiatry, Tokyo | Physiotherapist | Acquisition of physiotherapy data |
| Takahiro Nakayama MD PhD | Yokohama Rosai Hospital, Yokohama, Japan | Study Advisor | Study coordinator |
| Kazuhiko Segawa MD PhD | National Center of Neurology and Psychiatry, Tokyo, Japan | Study Doctor | Acquisition of medical history, medical and physical assessments |
| Makiko Endo | National Center of Neurology and Psychiatry, Tokyo, Japan | Study Coordinator | Study coordination and data management |
| Meganne E. Leach MSN, APRN | Children’s National Health System, Washington, DC, USA | Research Nurse | Acquisition of medical history, medical and physical assessments |
| Nora Brody PT, DPT | Children’s National Health System, Washington, DC, USA | Physiotherapist | Acquisition of physiotherapy data |
| Allyn Toles | Children’s National Health System, Washington, DC, USA | Study Coordinator | Study coordination and data management |
| Stanley T. Fricke PhD | Department of Diagnostic Imaging and Radiology, Children’s National Health System, Washington DC, USA | Radiologist | MRI data acquisition |
| Hansel J. Otero MD | Department of Diagnostic Imaging and Radiology, Children’s National Health System, Washington DC, USA | Radiologist | MRI data acquisition |
| Ulrike Grieben MD | Charite, Berlin, Germany | Study Doctor | Acquisition of medical history, medical and physical assessments |
| Juliana Prugel | Physio Plus, Berlin, Germany | Physiotherapist | Acquisition of physiotherapy data |
